# Cytological Studies of Human Meiosis: Sex-Specific Differences in Recombination Originate at, or Prior to, Establishment of Double-Strand Breaks

**DOI:** 10.1371/journal.pone.0085075

**Published:** 2013-12-20

**Authors:** Jennifer R. Gruhn, Carmen Rubio, Karl W. Broman, Patricia A. Hunt, Terry Hassold

**Affiliations:** 1 School of Molecular Biosciences and Center for Reproductive Biology, Washington State University, Pullman, Washington, United States of America; 2 Preimplantation Genetic Diagnosis Unit, Iviomics, Paterna, Valencia, Spain; 3 Department of Biostatistics and Medical Informatics, University of Wisconsin-Madison, Madison, Wisconsin, United States of America; Oklahoma Medical Research Foundation, United States of America

## Abstract

Meiotic recombination is sexually dimorphic in most mammalian species, including humans, but the basis for the male:female differences remains unclear. In the present study, we used cytological methodology to directly compare recombination levels between human males and females, and to examine possible sex-specific differences in upstream events of double-strand break (DSB) formation and synaptic initiation. Specifically, we utilized the DNA mismatch repair protein MLH1 as a marker of recombination events, the RecA homologue RAD51 as a surrogate for DSBs, and the synaptonemal complex proteins SYCP3 and/or SYCP1 to examine synapsis between homologs. Consistent with linkage studies, genome-wide recombination levels were higher in females than in males, and the placement of exchanges varied between the sexes. Subsequent analyses of DSBs and synaptic initiation sites indicated similar male:female differences, providing strong evidence that sex-specific differences in recombination rates are established at or before the formation of meiotic DSBs. We then asked whether these differences might be linked to variation in the organization of the meiotic axis and/or axis-associated DNA and, indeed, we observed striking male:female differences in synaptonemal complex (SC) length and DNA loop size. Taken together, our observations suggest that sex specific differences in recombination in humans may derive from chromatin differences established prior to the onset of the recombination pathway.

## Introduction

Although meiosis is essential for the production of both eggs and sperm, it is highly sexually dimorphic. Indeed, it could be argued that the differences between male and female meiosis are more numerous than the similarities. Oocytes initiate meiosis in the fetal gonad, enter an extended arrest phase (dictyate), and do not resume meiosis until the time of ovulation, some 10-50 years later [1]. During the female reproductive lifespan, the number of mature eggs produced is limited to several hundred and, because sperm penetration triggers the completion of the second meiotic division, only eggs that are successfully fertilized complete meiosis. In contrast, in the male, the onset of meiosis coincides with sexual maturation, germline stem cells ensure that large numbers of sperm are produced continuously, and the meiotic phase is comparatively short – approximately 3-4 weeks of the nine total weeks required to generate a mature sperm cell.

In addition to these obvious sex-specific temporal differences there is, particularly in humans, a striking difference in error rate between the sexes. An estimated 5-10% of clinically recognized human pregnancies are chromosomally abnormal, with the vast majority resulting in miscarriage or congenital birth defects [2]. The most common chromosome abnormality is trisomy, resulting from segregation errors during either of the two meiotic divisions. Studies of the origin of human trisomies demonstrate that nondisjunction can occur during either spermatogenesis or oogenesis, but that the overwhelming majority (75-90%) of trisomies result from errors during maternal meiosis [1–3].

Although the mechanisms responsible for the high rate of errors during female meiosis remain unclear, data from recent studies suggest that several factors, including meiotic protein degradation and checkpoint stringency play a role (reviewed in [4]). However, the only molecular mechanism that has yet been directly linked to human meiotic errors is one that occurs during the fetal stages of oogenesis: meiotic recombination.

Recombination is essential for the unique segregation of homologous chromosomes at the first meiotic division, and errors in recombination have been associated with nondisjunction in a variety of species [1,5,6]. In humans, failure to recombine and/or sub-optimally located crossovers have been linked to both paternally- and maternally-derived trisomies, but the association is particularly strong for cases of maternal origin [1,3,7–9].

Sex-specific differences in human meiotic recombination levels have been recognized for many decades [10–15], but we still have little understanding of the cause(s) of this variation. This is, in part, due to the limitations inherent in linkage analysis, which can only provide retrospective information on a small number of meiotic events per individual. Therefore, to better identify sex differences in the determination and establishment of meiotic recombination sites, we conducted a systematic comparison of human spermatocytes and oocytes throughout early prophase to determine the stage at which sex-specific differences first become apparent.

We combined immunofluorescence with fluorescence in-situ hybridization (FISH) to examine sex-specific differences in recombination in pachytene stage oocytes and spermatocytes. As a surrogate for recombination events, we scored the number of foci per cell for the DNA mismatch repair protein MLH1, thought to identify the vast majority of crossovers [6,16–18]. Our observations on MLH1 are consistent with previous linkage analyses [11–15], demonstrating significant increases in recombination in females, as well as sex-specific differences in the placement and spacing of recombination sites. To identify possible drivers of these sex-specific differences, we conducted comparative analyses of earlier stages of prophase. The differences in mid-prophase were correlated with differences in chromatin conformation as identified by synaptonemal complex (SC) length and DNA loop size. In addition, we observed differences in the number of DSBs as marked by RAD51 foci, and the number and placement of synaptic initiation sites. Taken together, our data suggest that sex-specific differences prior to or at the onset of DSB formation set the stage for sex differences in the pattern of recombination. We postulate that differences in chromatin compaction help establish these recombination differences, and that at least some of the vulnerability of human oocytes to recombination errors is a reflection of events that occur upstream of the formation of DSBs.

## Results

We used a cytological approach to directly examine meiosis in human male and female gametes, asking whether we could replicate the sex-specific differences in recombination observed in linkage studies and, if so, whether we could shed light on its basis. In total, we examined 4660 prophase spermatocytes from 56 testicular biopsies and 2038 prophase oocytes from 63 fetal ovaries; information on the individual patient samples used for these analyses is provided in Tables S1-S4.

### Genome-wide and chromosome-specific recombination rates are lower in males than in females

**Figure 1:**
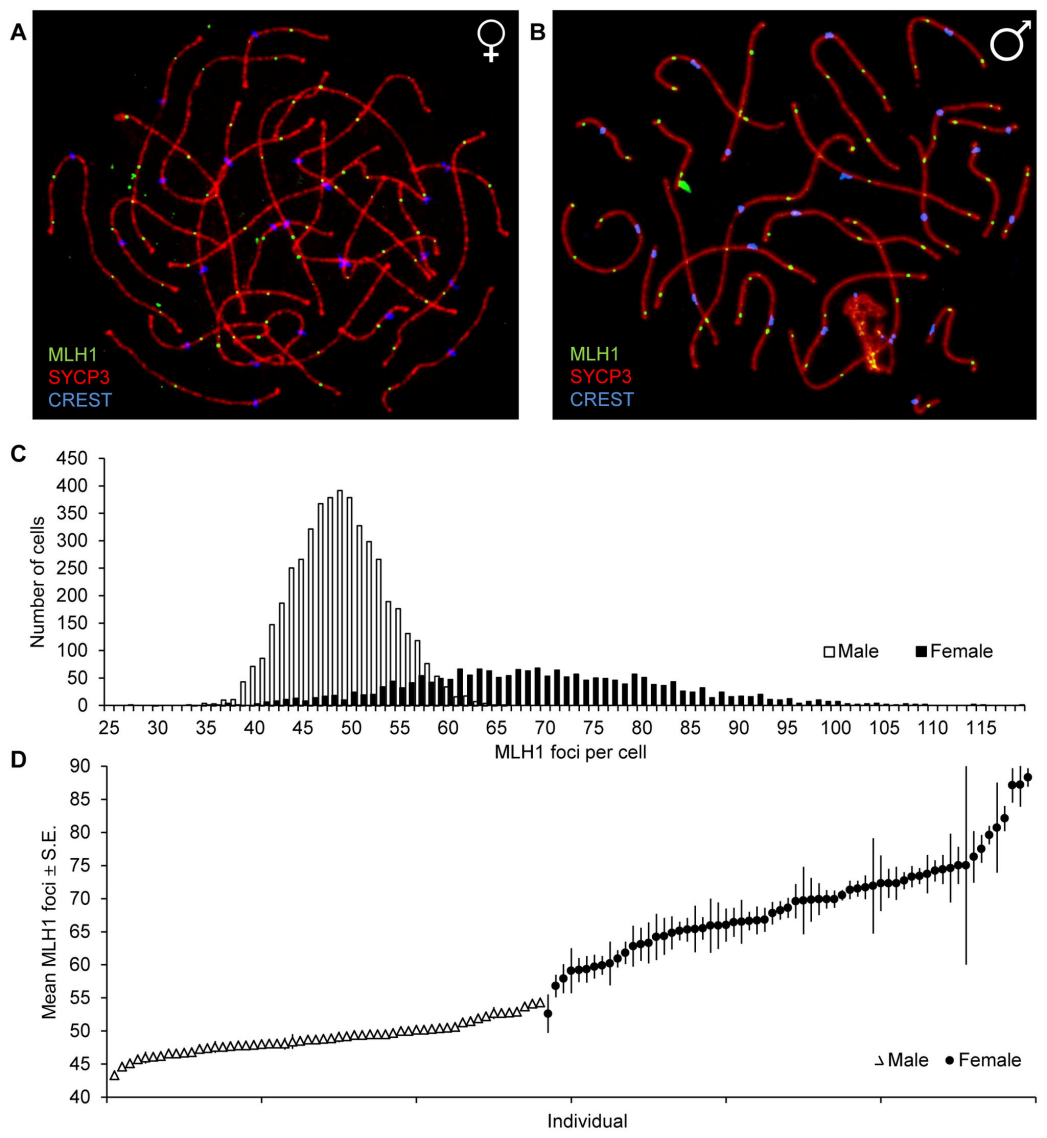
Genome-wide mean MLH1 values in human males and females. MLH1 foci were used as a marker for meiotic recombination events. (A) shows a representative pachytene oocyte and (B) a representative pachytene spermatocyte, that were immunostained for SYCP3 (in red), a component of the synaptonemal complex; CREST (in blue), detecting centromeric regions; and MLH1 foci (in green), detecting crossovers. (C) In total 4660 spermatocytes from 56 males (white) and 2038 oocytes from 63 females (black) were examined. Mean MLH1 counts (± S.E.) were significantly lower in males than females (49.09 ± 0.07 vs. 69.25 ± 0.29; t=92.5, p<0.0001) and the range was narrower in males than females (30-66 vs. 27-119). (D) Mean number (± S.E.) of MLH1 foci per cell for individual male and female samples, demonstrating the lack of overlap between the sexes, and the increased variation in individual female cases by comparison with males.

The DNA mismatch repair protein, MLH1 is an established marker of the vast majority of meiotic recombination events [6,16–18], and was used to estimate the number of crossovers in human oocytes and spermatocytes (Figure 1A and 1B). As predicted from human linkage data, our analysis of genome-wide recombination rates revealed highly significant differences in mean values. The mean MLH1 foci per cell (± S.E.) was 49.09 ± 0.07 in males (n = 4660 spermatocytes) and 69.25 ± 0.29 in females (n = 2038 oocytes) (t=92.5; p<0.0001; Figure 1C). These observations are similar to previous MLH1 analyses of smaller series of human males or females (e.g., [19–27]), as well as to comparisons of males and females in large-stage linkage analysis [11–15]. However, unlike linkage studies, the cytological approach allows for the analysis of recombination in many cells per individual, and our observations provide important evidence that the range of MLH1 values is dependent on sex. Males displayed a much narrower range, from 30 to 66 MLH1 foci per cell, while in females cells had as few as 27 and as many as 119 foci. The wide variation in MLH1 values was also seen among individuals of the same sex, with means ranging from 43.30 to 54.29 in males (Table S2; Figure 1D) and from 52.62 to 88.25 in females (Table S4; Figure 1D).

**Figure 2:**
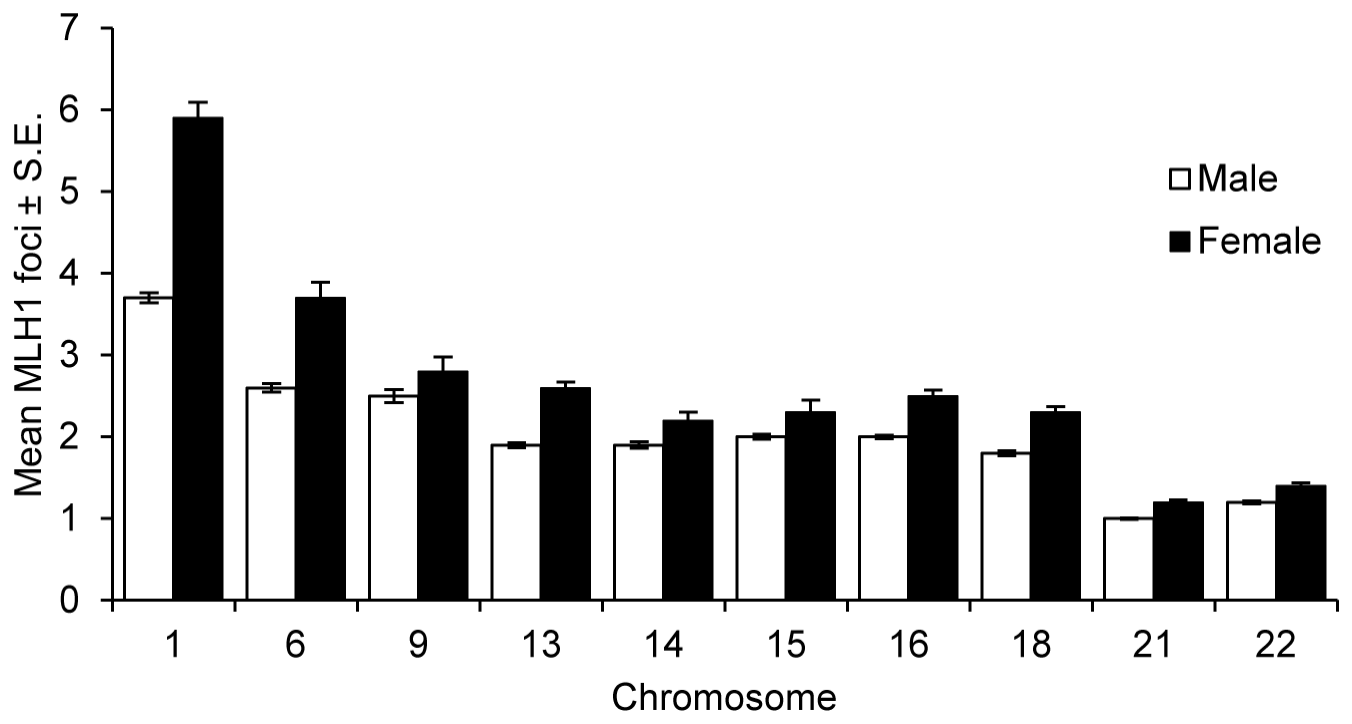
Chromosome-specific MLH1 values in males and females. Ten representative large, medium and small chromosomes were identified by FISH and chromosome-specific MLH1 values determined. For all ten chromosomes, mean values were lower in males (white) than females (black), and for nine of the ten the differences were statistically significant: chromosome 1 (10 males, n of cells =139; 7 females, n=83; t=14.1; p<0.0001), 6 (5 males, n=174; 3 females, n=30; t=8.2; p<0.0001), 13 (4 males, n=139; 8 females, n=109; t=8.4; p<0.0001), 14 (4 males, n=142; 4 females, n=70; t=3.2; p<0.005), 15 (6 males, n=130; 3 females, n=45; t=3.3; p<0.005), 16 (12 males, n=204; 9 females, n=63; t=8.8; p<0.0001), 18 (2 males, n=187; 7 females, n=100; t=6.9; p<0.0001), 21 (10 males, n=302; 11 females, n=218; t=7.1; p<0.0001), and 22 (10 males, n=313; 11 females, n=161; t=4.4; p<0.0001). The difference did not reach significance for chromosome 9 (5 males, n=58; 2 females, n=34; t=1.9; p=0.064).

To determine if all chromosomes share this sex-specific difference in recombination, or whether a sub-set of chromosomes disproportionately contributes to the effect, we examined ten representative large, medium and small chromosomes (i.e., 1, 6, 9, 13, 14, 15, 16, 18, 21, and 22). As is evident from Figure 2, the magnitude of the male:female difference in recombination was similar for all chromosomes. For nine of the ten (chromosomes 1, 6, 13, 14, 15, 16, 18, 21 and 22), the difference was statistically significant; it did not reach significance for chromosome 9, presumably reflecting the relatively small number of cells analyzed (see Figure 2 legend for statistical results).

As expected, there was also a marked difference in exchange frequency between the sexes. For example, chromosome 1 had as few as two and as many as six exchanges in males, but as few as two and as many as ten exchanges in females. The frequency of bivalents lacking an MLH1 focus also differed significantly, i.e., 0.38% in males vs. 2.60% in females, respectively (χ^2^=35.9; p<0.0001) (Table S5).

### MLH1 foci are preferentially distally-placed in males and interstitially-placed in females

**Figure 3:**
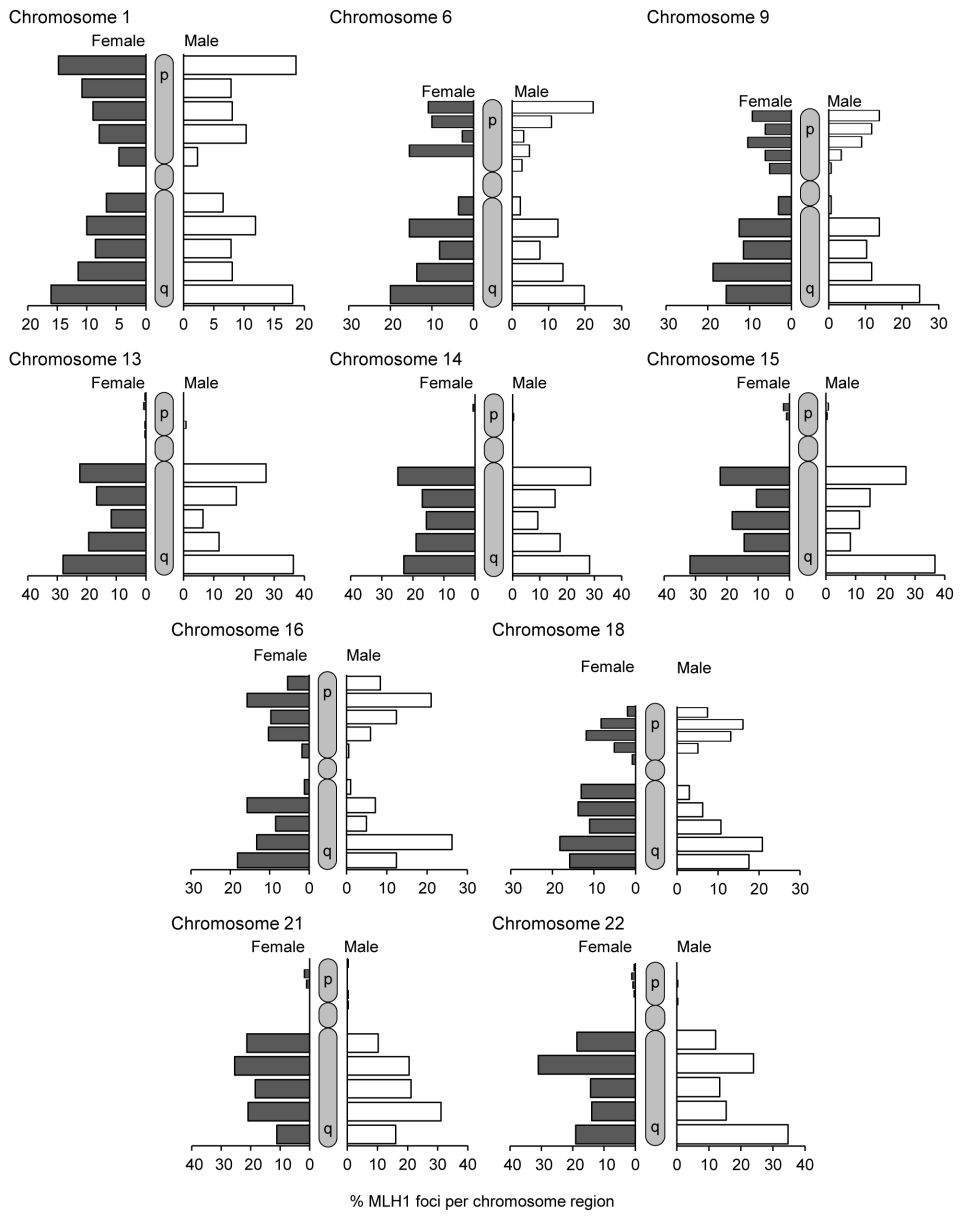
Chromosome-specific MLH1 localization patterns in males and females. The chromosomal locations of MLH1 foci were determined using the same cells as in Figure 2 for ten representative large, medium and small chromosomes. Each chromosome arm was arbitrarily divided into five equal regions – centromeric, proximal, interstitial, distal, and telomeric – and the distribution of MLH1 foci recorded for both chromosome arms for metacentric and sub-metacentric chromosomes or for the q-arm only of acrocentric chromosomes. The distribution differed significantly between females (black) and males (white) for seven of the ten chromosomes: 1 (χ^2^=24.9; p<0.005), 6 (χ^2^=24.8; p<0.005), 13 (χ^2^=13.8; p=0.01), 16 (χ^2^=32.1; p<0.0001), 18 (χ^2^=47.7; p<0.0001), 21 (χ^2^=22.3; p<0.0001), and 22 (χ^2^=20.8; p<0.0001). However, sex-specific differences were not evident for chromosomes 9 (χ^2^=15.1; p=0.088), 14 (χ^2^=5.3; p=0.262), or 15 (χ^2^=7.8; p=0.101).

In addition to determining their number, we determined the approximate location of MLH1 foci for the same ten representative chromosomes (Figure 3). For this analysis, each chromosome arm was divided into five equal segments: centromeric, proximal, interstitial, distal, and telomeric. For most chromosomes, males had a higher proportion of distally-placed MLH1 foci and females had a preponderance of interstitially-placed foci. Statistically significant sex-specific differences in MLH1 placement were seen on the p- and q-arms of chromosomes 1, 6, 16, and 18 and the q-arms of chromosomes 13, 21, and 22; significant sex-specific differences were not noted for chromosomes 9, 14, and 15 (see legends of Figures 3 for statistical results).

### Males display increased spacing of MLH1 foci

**Figure 4:**
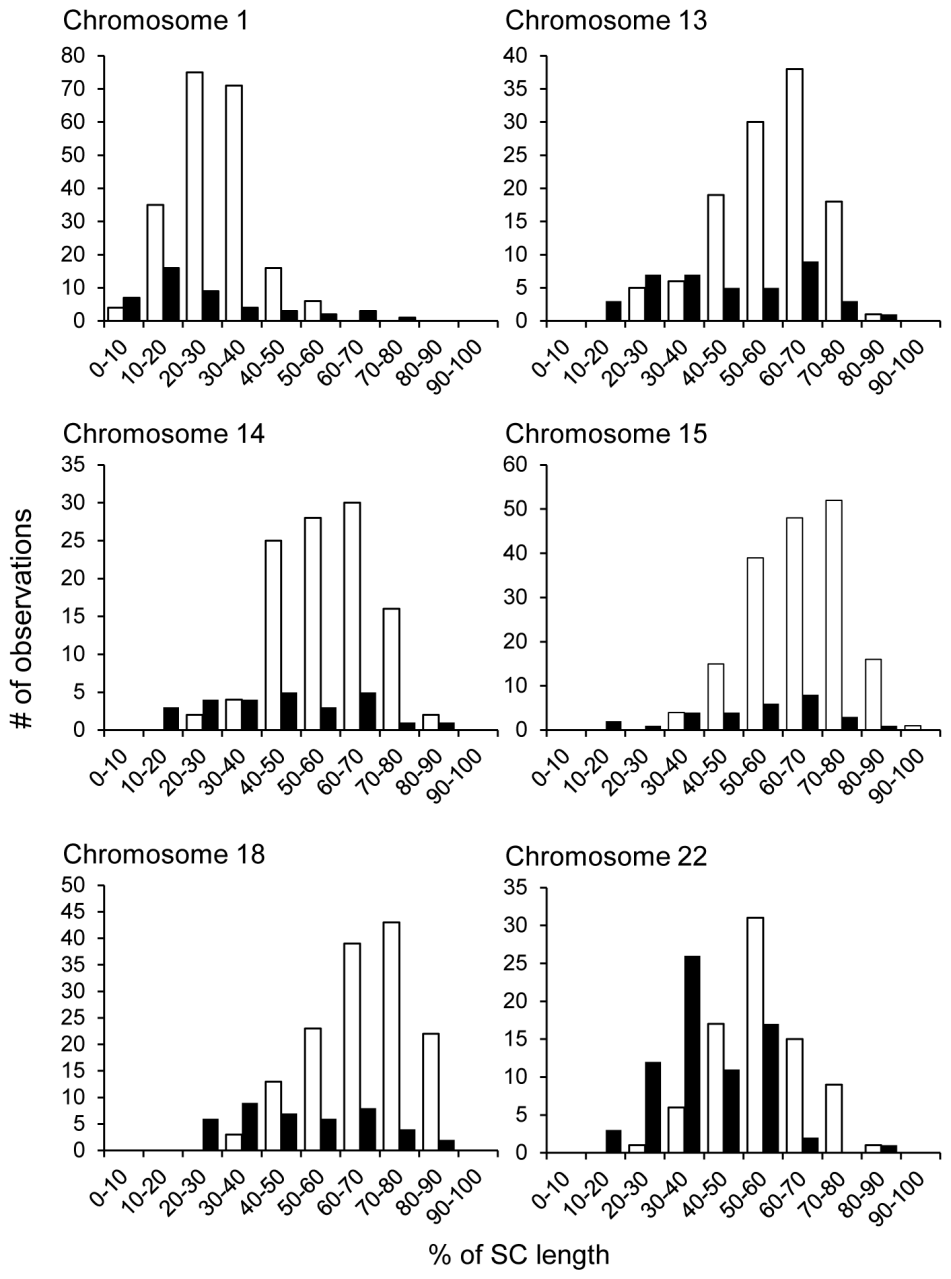
Spacing between adjacent MLH1 foci in males and females. Inter-focal distances, calculated as the percent of the length of the synaptonemal complex between adjacent MLH1 foci, were determined using the same cells as in Figure 2; male data are depicted in white, female data in black. To obtain sufficient numbers of cells for direct male:female comparisons, we restricted our analysis to chromosomes having the same number of MLH1 foci in males and females; i.e., for chromosome 1 we analyzed cells in which the chromosome exhibited four MLH1 foci and for chromosomes 13, 14, 16, 18 and 22, cells in which the relevant chromosome exhibited two MLH1 foci. Thus, for chromosome 1, we made three measurements of inter-focal distances per cell, while for chromosomes 13, 14, 16, 18 and 22 we made a single measurement of inter-focal distance per cell. For chromosomes 6, 9, 15 and 21 we had a limited number of cells with the same number of MLH1 foci in both sexes; thus, these chromosomes were excluded from the analysis. For each chromosome, inter-focal distances were binned (by % value) into ten groups. The distribution of categories of inter-focal distances was significantly different between males and females for each of the six chromosomes: 1 (χ^2^=51.7; p<0.0001), 13 (χ^2^=26.7; p<0.0005), 14 (χ^2^=30.6; p<0.0001), 16 (χ^2^=31.9; p<0.0001), 18 (χ^2^=50.0; p<0.0001), and 22 (χ^2^=48.8; p<0.0001).

To identify possible sex-specific differences in the spacing of exchanges, we examined chromosomes with multiple MLH1 foci. We restricted our analyses to chromosomes 1, 13, 14, 16, 18 and 22, since at least 25 observations per sex were available on each of these chromosomes. For chromosomes 13, 14, 16, 18 and 22 we examined chromosomes with two MLH1 foci, calculating the inter-focal distance as a proportion of the total SC length; for chromosome 1, which had a higher mean number of MLH1 foci, we restricted our analysis to chromosomes with four MLH1 foci. For all six chromosomes, we binned the inter-focal distances into ten equal groups and compared the distributions between males and females (Figure 4). The distributions were significantly different for each of the six chromosomes (see Figure 4 legend for statistical results), and were attributable to increased inter-focal distances in males by comparison with females.

### Analysis of SC length and DNA loop size indicates male:female differences in chromatin conformation at pachytene

**Figure 5:**
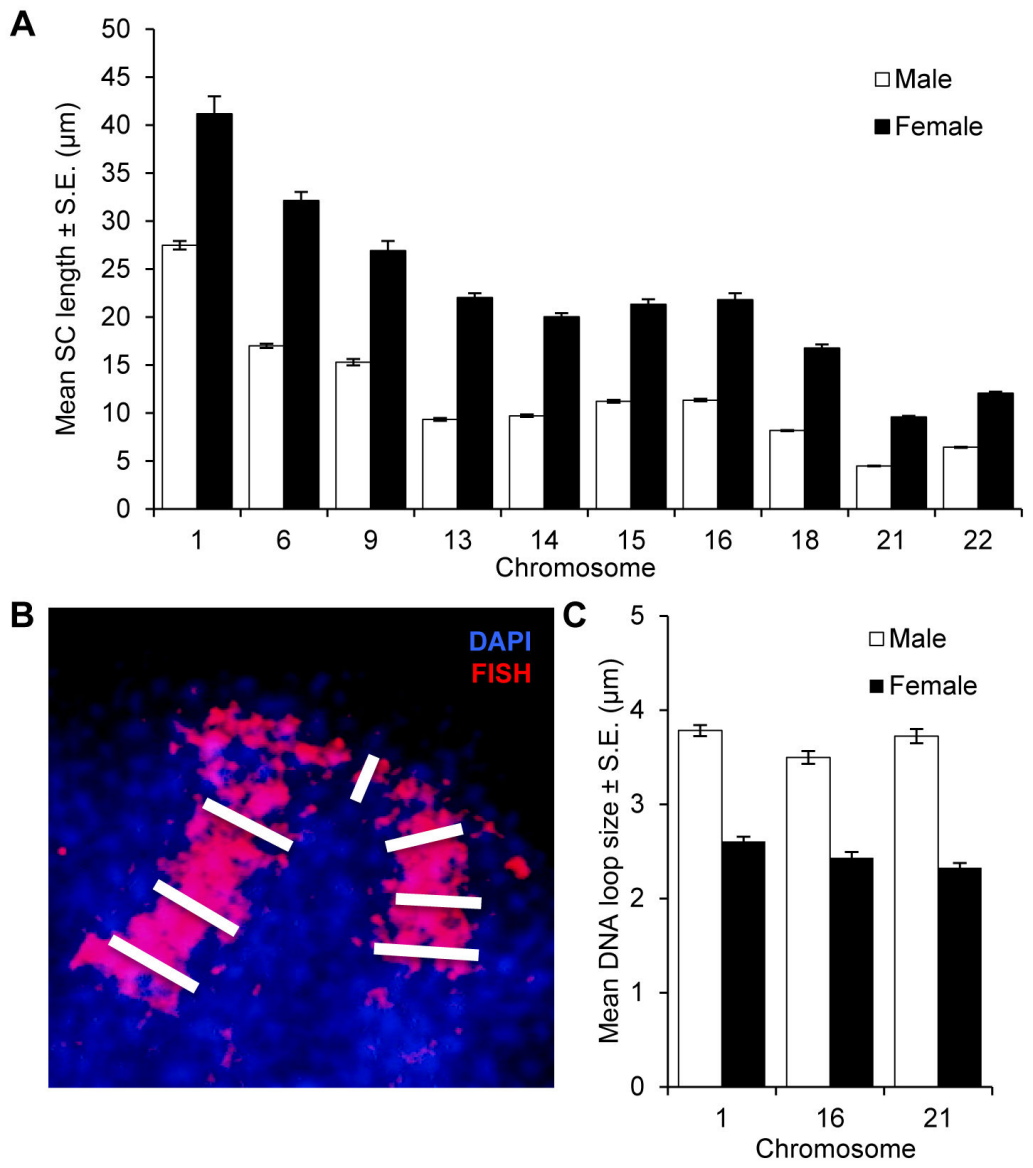
Comparison of SC length and DNA loop size in males and females. Male data are in white, female data in black. (A) Chromosome-specific SC lengths were determined for cells scored in Figure 2 and striking sex-specific differences were evident on all ten chromosomes analyzed: 1 (t=8.9; p<0.0001), 6 (t=23.1; p<0.0001), 9 (t=12.8; p<0.0001), 13 (t=26.7; p<0.0001), 14 (t=30.8; p<0.0001), 15 (t=24.1; p<0.0001), 16 (t=21.7; p<0.0001), 18 (t=28.6; p<0.0001), 21 (t=36.4; p<0.0001), and 22 (t=35.4; p<0.0001). (B, C) Three individual chromosomes (1, 16 and 21) were analyzed for DNA loop size, using the deflection of FISH paint probes from the SC as a surrogate for loop size. For chromosomes 1 and 16, we measured the width of the FISH signal at the centromere and three points on each chromosome arm, and averaged the seven values. For chromosome 21, loop size was taken as the average of three measurements, one at the centromere and two on the long arm. (B) Blow-up image of a portion of a representative pachytene stage oocyte, labeled with DAPI (blue) and a chromosome 1 paint probe (red). White bars represent the seven individual DNA loop measurements, three from each chromosome arm and one at the centromere. The centromere was identified using CREST prior to FISH. (C) DNA loop size means were significantly greater in males for each chromosome; i.e., for chromosome 1 (2 males, n of cells=24; 3 females, n=23; t=15.2; p<0.0001), for 16 (2 males, n=39; 3 females, n=32; t=20.8; p<0.0001) and for 21 (2 males, n=37; 2 females, n=43; t=16.0; p<0.0001).

Previous studies by us [28] and others [19] have suggested an association between the length of the synaptonemal complex and the number of MLH1 foci. Accordingly, we compared genome-wide SC lengths, defined as the sum of the lengths (in microns) of the SYCP3 signals, between males and females. Consistent with immunofluorescence and electron micrographic analyses of SCs in males and females (e.g, [29,30]), the mean genome-wide SC length was significantly reduced in males by comparison with females (293.87 ± 11.23 and 609.10 ± 31.86, respectively; t=11.4; p<0.0001). Subsequently, we conducted similar analyses of the same 10 individual chromosomes analyzed for MLH1 foci and found that, for each chromosome, male SC lengths were significantly shorter than female (Figure 5A; see Figure 5 legend for statistical results).

Subsequently, we compared DNA loop sizes between males and females, using chromosome-specific paint FISH probes to chromosomes 1, 16 and 21 as surrogates for the DNA loops (Figure 5B). In total, we analyzed 77 pachytene stage cells from three males and 98 cells from seven females; for each chromosome, we recorded the average of multiple measurements of the width of the FISH signals. The results were consistent across all three chromosomes, with males displaying significantly larger DNA domains than females (Figure 5C; see Figure 5 legend for statistical results). Taken together, the analyses of SC lengths and DNA loop sizes indicate marked differences between males and females in the way in which DNA is packaged on the SCs*.*

### Consistent with sex-specific MLH1 differences, males form fewer double-strand breaks than females

**Figure 6:**
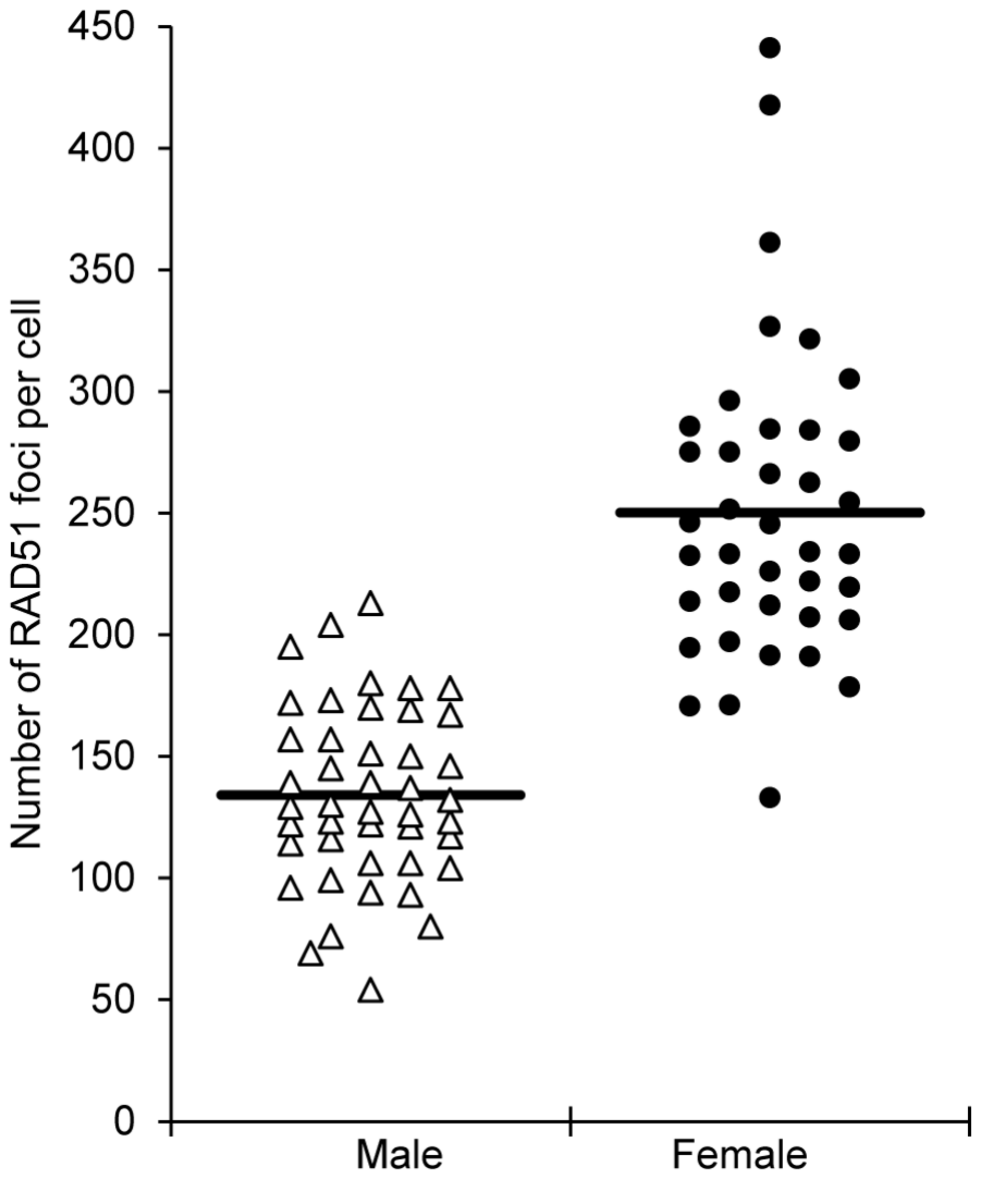
Genome-wide RAD51 values in males and females. RAD51 foci were used as a surrogate for DSBs and the number of foci in leptotene stage cells determined [44 cells from 3 males (white triangles) and 39 cells from 5 females (black circles)]. The male and female distributions were virtually non-overlapping, with almost all spermatocytes exhibiting fewer than 200 RAD51 foci and most oocytes more than 200 foci. Further, the mean number of RAD51 foci (± S.E.) was significantly lower in males than in females (134.07 ± 5.47 and 250.28 ± 10.21, respectively; t=10.4; p<0.0001).

Sex-specific variation in recombination levels could originate at several steps in the recombination pathway; e.g., from differences in the number of DSBs, in the initial processing of DSBs, or in the resolution of double Holliday junctions as crossovers or non-crossovers [31]. To investigate the earliest of these events, we asked whether the number of DSBs differed in males and females. We used the homology recognition and DNA strand exchange protein RAD51, a marker of SPO11-initiated DBSs [32], to estimate the number of DSBs in leptotene and zygotene stage spermatocytes and oocytes. A comparison of genome-wide DSB numbers between the sexes revealed a highly significant difference in mean values; i.e., the mean number of RAD51 foci per cell (± S.E.) was 134.07 ± 5.47 (n = 44 spermatocytes) and 250.28 ± 10.21 (n = 39 oocytes) for males and females, respectively (t=10.4; p<0.0001) (Figure 6). This is consistent with previous immunofluorescence analyses of RAD51 in human males [33,34] and females [22] and suggests that sex-specific recombination differences are already in place when the recombination pathway is initiated.

### The initiation of synapsis differs between males and females

In simple eukaryotes such as yeast, a direct link has been made between sites of synaptic initiation between homologs and sites of recombination (e.g., [35,36]). Consequently, we asked whether the male:female differences we observed in the localization of MLH1 foci were reflected by similar sex-specific differences in sites of synaptic initiation.

**Figure 7:**
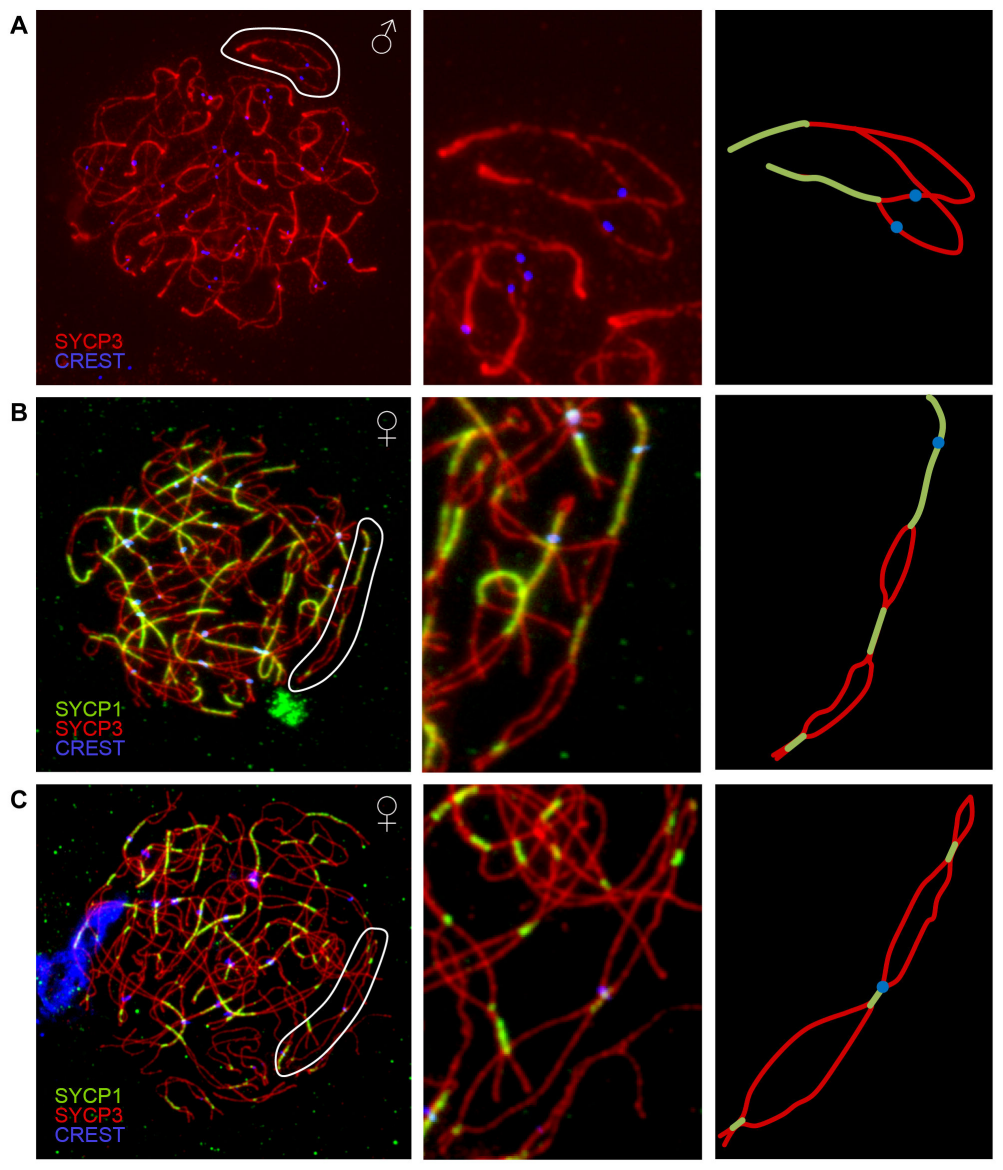
Synaptic initiation complexes (SCISs) in zygotene meiocytes. (A) Representative zygotene spermatocyte (left), with antibodies against SYCP3 (detecting the axial/lateral elements of the SC) in red and CREST antiserum-positive signals (recognizing centromeric regions) in blue. Intense red signals indicate points where the axial/lateral elements have merged, consistent with full synapsis (i.e., SCISs). SCISs are located at, or near the telomeres, an arrangement typical for human males. The center panel shows a blow-up of a partially-synapsed bivalent (circled) and the right panel provides a schematic of the bivalent, with the synapsed regions at the ends of the arms and the proximal regions (including the centromeres) asynapsed. (B, C) Representative images of zygotene stage oocytes (left), with the axial/lateral element protein SYCP3 in red, the transverse element protein SYCP1 in green and centromere-associated CREST in blue; merged SYCP3/SYCP1 signals (yellow) indicate regions of synapsis (i.e., SCISs). Center panels provide blow-ups of partially synapsed bivalents (circled) and the right panel schematics, demonstrating the presence of multiple SCISs per chromosomes and in (C), co-localization of the centromere and one of the SCISs.

A synaptonemal complex initiation site (SCISs) can be examined by monitoring localization patterns of the transverse element protein SYCP1 in zygotene stage cells. In previous studies of human males, we identified three virtually invariant features of SCISs: there were two SCISs on non-acrocentric chromosomes, one on each arm, and one SCIS (on the q arm) on acrocentrics; SCISs were located near the telomeres; and synapsis proceeded by “zippering-up” from distal chromosome regions toward the centromere, which was typically the final chromosome region to synapse (Figure 7A) [29]. To determine whether these rules also applied to human females, we examined SCISs in 120 zygotene cells from two individuals. Our observations of SYCP1 localization indicated a remarkably different pattern of synaptic initiation in females. SCISs typically occurred at multiple sites along each chromosome (Figure 7B); e.g., in chromosome specific analyses, the mean number of SCISs per bivalent was 2.16 ± 0.13 (± S.E.) for chromosome 16, with as many as six SCISs on a chromosome, and 1.60 ± 0.06 for chromosome 21, with as many as three on a chromosome. Further, SCISs were not necessarily telomeric; indeed, interstitial and pericentromeric locations predominated. Additionally, unlike the male, the centromere did not seem to be refractory to SCISs (Figure 7C). Indeed, in an analysis of a subset of 15 cells containing 310 partially synapsed bivalents, nearly one-half (129/310 = 41.6%) of all SCISs included the centromere. Taken together, these observations mirror the sex-specific differences noted for recombination since – as was observed for MLH1 foci – females had an increased number of SCISs, and SCISs were more likely to be interstitially or proximally located.

## Discussion

Human females are extraordinarily prone to nondisjunctional errors in meiosis, with an estimated 10-30% of fertilized eggs having too few or too many chromosomes [1]. Conventional wisdom attributes this to the protracted lifecycle of the human oocyte, implicating errors occurring during the meiotic arrest phase or during the final stages of oogenesis when the egg is “re-booted” and enters the meiotic divisions. Consistent with this idea, the frequency of maternally-derived aneuploidy increases exponentially in pregnancies involving women over 35 years of age [1].

However, there are at least two lines of evidence indicating that events occurring at the earliest, fetal, stages of oogenesis also contribute to the genesis of human aneuploidy. First, studies of trisomic conceptions link abnormalities in meiotic recombination – a process that takes place in the fetal oocyte – to the origin of maternally-derived cases of trisomies 13, 15, 16, 18, 21, 22 and sex chromosome trisomies [1,37]. Second, recent cytological studies of human pachytene oocytes provide direct evidence for a high proportion of cells with non-recombinant bivalents, indicating that some oocytes are, indeed, predisposed to nondisjunction from the beginning stages of meiosis [21].

These observations raise an obvious question: Could the well-known excess of maternally-derived trisomies be attributable – at least in part – to sex-specific differences in patterns of meiotic recombination established at the earliest stages of oogenesis and spermatogenesis? Accordingly, we set out to characterize basic features of recombination in males and females, taking a cytological approach to examine early prophase spermatocytes and oocytes to gain insight into the origin of the male:female variation in recombination.

### Genome wide recombination rates in males and females: what cytology can tell us

Our cytological analyses of recombination, using MLH1 as a surrogate for crossovers [6,16–18], differ from previous linkage studies [11–15] in one important respect, namely the overall rate of recombination. Assuming that one crossover (MLH1 focus) = 50 cM, our observations suggest genome wide genetic lengths of 3463 cM in females and 2455 cM in males. In contrast, recent linkage analyses (e.g., [14]) report female maps of approximately 4500-4600 cM, some 1.3 fold longer than ours, and a male map of approximately 2800-2900 cM, 1.1-1.2 fold in excess of ours. Importantly, our cytological approach captures only MLH1-associated crossovers, but as many as 10% of crossovers are thought to involve MLH1-independent pathways [17,18]. While this accounts for most, if not all, of the difference between the cytological and linkage-based male maps, it does not explain the magnitude of the difference between the MLH1 and linkage maps for the female. Here we suspect that a unique feature of human female meiosis plays a role. That is, our previous cytological studies of human females [21] indicate that MLH1 localization occurs over a wide temporal window, suggesting that the analysis of MLH1 foci in pachytene stage cells may not capture all MLH1-mediated recombination events. Since this does not appear to be the case for human males (Hassold, unpublished observations), only the female map would be affected.

Despite the discrepancy between cytological and linkage maps with respect to the overall rate of recombination, the maps share two important features – a highly significant excess of recombination in the female, and sex-specific differences in the placement of exchanges. These results are consistent with analyses from a variety of mammalian species, which typically indicate overall increases in recombination in females by comparison with males [10,38–40]. Thus, we conclude that the cytological approach provides a useful, direct approach to the analysis of the vast majority of recombination events in humans.

To investigate the basis of the sex-specific differences, we asked whether we could identify other sexually dimorphic features of meiotic prophase. Of particular interest was the possibility that the male:female variation might be evident at the beginning of the recombination pathway, as reflected by differences in the number of DSBs. Accordingly, we analyzed RAD51 foci in early prophase meiocytes, and observed nearly twice as many foci in females. We scored only spherical, well-spaced and intensely staining foci, and thus almost certainly underestimated the “real” RAD51 values in both spermatocytes and oocytes [22,33,34]. Further, we made no attempt to analyze other, later-occurring recombination intermediates (e.g., by scoring the number of MSH4 or MSH5 foci), and cannot be certain that the male:female differences we observed for RAD51 and MLH1 are evident at these intermediate stages. Nevertheless, our observations provide strong evidence that, at the onset of meiosis, there are more DSBs in human females than in males. Assuming that a similar proportion of breaks are processed into crossovers in spermatocytes and oocytes, we would expect to visualize more MLH1 foci in females than males, a prediction consistent with our observations. Thus, we suggest that the underlying mechanisms responsible for sex-specific variation in crossing-over are already present at the time of DSB formation.

We were also interested in determining whether the way in which homologous chromosomes synapse is different in males and females. The formation of the mature SC is thought to be dependent on DSBs and studies of several model organisms have shown a correlation between sites of synaptic initiation and the location of crossovers [35,36,41,42]. Consistent with these reports, we detected a similar relationship between MLH1 foci and SCISs in previous analyses of human males [29]. Specifically, in our analyses of zygotene spermatocytes, SCISs were predominantly distally located on chromosomes, mimicking the distribution of MLH1 foci in pachytene cells. The relationship between SCISs and MLH1 foci was not exact, however, since there were more MLH1 foci than SCISs and, in general, SCISs appeared to be more distally positioned on the chromosome arms than were MLH1 foci ([29] and Hassold, unpublished observations). Nevertheless the localization patterns were strongly correlated and, accordingly, we were interested in asking whether there was a similar relationship between SCISs and MLH1 foci in human females. The results of initial studies reported here suggest that the answer is yes. Like MLH1 foci, we identified multiple SCISs per chromosome in oocytes, with interstitial and proximal locations predominating. Additional mapping studies are required to determine whether SCISs and MLH1 foci co-localize/overlap or whether, as in males, there is simply a general correlation. Nevertheless, in the context of male:female meiotic differences the conclusion is clear, sex-specific differences are not restricted to recombination, but are also evident in the way that homologous chromosomes associate with one another.

In addition to the differences in MLH1 values and SCISs, our observations indicate sex-specific variation in the way in which the DNA is packaged during meiotic prophase. Fully formed SCs were approximately twice as long in females as in males (Figure 5A), while the sizes of the DNA loops emanating from the SC were significantly larger in males than in females (Figure 5C). Because these measurements required analyses of fully formed SCs, they were restricted to the stage of meiotic prophase at which all homologous chromosomes are fully synapsed; i.e., pachytene. Since DSB formation and the initiation of synapsis occur much earlier in prophase, it remains unclear whether differences in chromatin configuration are established prior to the onset of meiosis or during early prophase when the recombination pathway is initiated. However, we favor the former, since observations of FISH images from leptotene stage cells indicate similar sex-specific differences in DNA domain size as those visualized in pachytene preparations (Figure S1).

In summary, our analyses of different markers of recombination (i.e., MLH1, RAD51), synapsis (number and location of SCISs) and chromatin morphology (SC length and DNA loop size) were consistent in identifying sex-specific differences. The magnitude of the effects was somewhat variable; e.g., on average, females had 1.4 fold as many MLH1 foci as males, but 1.9 fold as many RAD51 foci, while the genome-wide SC length in females was approximately 2 fold that of males. Nevertheless, in each instance the differences were highly significant, indicating sex-specific variation in the way that homologs interact with one another in meiotic prophase.

### How can upstream meiotic prophase events generate male:female differences in recombination?

Taken together, our analyses suggest that sex-specific differences in genome wide recombination levels are shaped by events that occur at, or before, the initiation of recombination pathway per se. To date, only three loci – *RNF212*, *PRDM9* and a common inversion at chromosome 17q21.31 – have been linked to variation in recombination levels and/or recombination hotspots in humans, and it is of interest to ask whether of any these might play a role in mediating the male:female recombination differences.

*RNF212*, encoding a homolog of the crossover-associated proteins Zip3 in *S. cerevisiae* and ZHP-3 in *C. elegans*, is perhaps the most attractive candidate. In an analysis of an Icelandic population, Kong et al [43] linked sequence variation at *RNF212* to individual differences in genome-wide recombination levels in both males and females; i.e., in each sex, the presence of a single copy of a “high” haplotype at *RNF212* increased recombination by approximately 70-80 cM over that of a “low” haplotype. Subsequent analyses of other populations have confirmed the association between *RNF212* and genome-wide recombination rates in males [44] or in both sexes [45]. Thus, it is clear that *RNF212* is an important regulator of overall levels of recombination in both human males and females. However, the importance of *RNF212* to differences between males and females is not as obvious. The polymorphic differences at *RNF212* explain only a small fraction of the observed variation in genome-wide recombination levels, and are insufficient to account for the much larger differences between the sexes. More importantly, a recent study of *Rnf212* in the mouse [42] suggests that it functions downstream of DSBs in stabilizing the crossover intermediate MSH4 and not at, or before, formation of DSBs. Thus, we suggest that, while RNF212 clearly plays a role in generating variation within males or females, it likely does not account for the differences between the sexes. Similarly, *PRDM9* and the chromosome 17 inversion also seem unlikely to explain sex-specific differences in recombination. The histone methyltransferase PRDM9 is important in the specification of recombination hot spots in mammals, but allelic variation appears to have little effect on genome-wide recombination rates in humans (e.g., [45]). Similarly, haplotype differences at the 17q21.31 inversion locus have only a minor effect on recombination rates (up to a 1% increase in recombination for the H2 haplotype) and have only been documented for females [46]. Thus, we suggest that other, as yet unidentified, loci are responsible for the male:female differences.

What processes might these loci affect? One obvious possibility is the programming of epigenetic marks, since this impacts chromatin structure and occurs at different developmental time points in the life cycles of mammalian male and female germ cells. Primordial germ cells (PGCs) undergo global demethylation as they proliferate and migrate to the genital ridge [47,48]; e.g., in the mouse, global methylation levels are under 20% for both male and female PGCs at embryonic day 13.5 [49,50]. Females enter meiosis immediately thereafter, thus oocytes proceed through the earliest stages of prophase in a demethylated state. In contrast, male germ cells enter into a quiescent mitotic arrest phase and do not undergo meiotic entry until after birth, by which time epigenetic reprogramming is nearly complete, and the genome is remethylated to levels over 75% [50–52]. Thus, the recombinational process – from the initiation of DSBs to the generation of genetic exchanges – is accomplished under strikingly different methylation states in mammalian spermatocytes and oocytes.

There is relatively little information from mammals on the effects that such variation in methylation might have on recombination levels, but evidence from model organisms indicates a strong correlation. For example, in the fungus *Ascobolus*, methylation of a recombination hotspot has been shown to dramatically reduce recombination levels between markers flanking the locus [53]. Further, several recent reports suggest an effect of methylation on recombination in *Arabadopsis* [54–57]. Specifically, analyses of mutants that decrease methylation levels indicate an increase in recombination in euchromatic chromosome regions, suggesting an association between hypomethylation and accessibility of DNA to recombination-promoting protein complexes. The effect depends on genomic context, with differential effects on pericentromeric vs. distal sequences, and with relatively little effect on the overall number of crossovers. Nevertheless, the results provide strong evidence that methylation can act to change the recombination landscape of the *Arabadopsis* genome.

Assuming that these effects extend to mammals, differences in the methylation states of prophase stage spermatocytes and oocytes may well contribute to the sex-specific differences in recombination levels. Indeed, in a recent analysis of female mice homozygous for a null mutation in the demethylating gene *Tet1*, defective demethylation for a subset of meiotic genes was linked to reduced numbers of MLH1 foci [58]. This does not mean that the methylation state is the sole mediator of recombination levels. Repressive and permissive histone marks are generally correlated with hyper- and hypo-methylation levels, respectively, and it seems likely that these and other epigenetic modifications work synergistically to “set” recombination levels. Nevertheless, we suggest that methylation is an important determinant of the recombination landscape in mammals, and of the sex-specific differences observed in many species. By manipulating methylation levels in PGCs, it should be relatively straightforward to test this idea. For example, mutations for a number of demethylating (e.g., *Tet* and *Dnmt* family members) and methylation-promoting loci (e.g., *Mthfr*), are available in mice, and by directly comparing MLH1 values in oocytes and spermatocytes of animals with different genotypes – especially wildtype and heterozygotes, not just null animals – it should be possible to determine whether there is a correlation between methylation levels. If so, additional studies can be directed at determining the temporal origin of the effect and, whether, similar to the results described in the present report, it arises at or before the formation of DSBs.

## Materials and Methods

### Ethics statement

This study was conducted according to the principles expressed in the Declaration of Helsinki. Initial results on a subset of study participants were previously reported [21,23], and the consent procedures were outlined in those manuscripts. For all new study participants, procedures were reviewed and approved by the Instituto Valenciano de Infertilidad, University of California-San Francisco and Washington State University Institutional Review Boards, and informed written consent was obtained from all study participants.

### Sample population

The sample population consisted of 4660 prophase spermatocytes from 56 testicular biopsies and 2038 prophase oocytes from 63 fetal ovaries; information on the individual cases is provided in Tables S1 and S3. Testicular biopsies were obtained from 54 patients being seen for infertility at either the Urology Department of the University Hospitals of Cleveland (UH), Cleveland OH, the Glickman Urological Institute of the Cleveland Clinic Foundation (CCF), Cleveland, OH or the Instituto Valenciano de Infertilidad (IVI), Valencia, Spain, and from two UH patients following surgeries for testicular tumors. Patient ages ranged from 30-80 years; analyses of infertile patients were restricted to individuals diagnosed with obstructive azoospermia, typically attributable to a previous vasectomy. Initial results on a subset of cases ascertained at UH or CCF were presented previously [23].

Fetal ovaries were obtained from elective or therapeutic terminations of pregnancy performed at either the University of Washington Medical Center, Seattle, Washington or at the San Francisco General Hospital Women’s Options Center in San Francisco, California. Gestational ages for the samples ranged from 14-25 weeks gestation. Initial results on a subset of cases were presented previously [21].

### Tissue processing

Material was collected from testicular or fetal ovarian tissues and processed using a standard surface-spreading technique [59]. For male samples, tissue from seminiferous tubules was macerated and incubated in hypotonic solution for approximately one hour, and immunostaining performed within 24 hours of slide preparation as previously described [60]. For female samples, fetal ovaries were isolated and excess connective tissue removed, the ovaries placed in a hypo-extraction buffer and subsequently macerated and spread onto microscope slides for immunostaining the following day [21].

### Immunostaining

Slides were immunostained using similar methodology to that of Cheng et al. [21]. Antibodies were diluted in sterile filtered 1xADB consisting of 10 ml normal donkey serum (Jackson ImmunoResearch), 3 g BSA (Sigma-Aldrich), 50 μl Triton X-100 (Alfa Aesar), and 990 ml PBS. Incubations were performed in a dark humid chamber at 37°C.

Slides were first blocked in 1x ADB for one hour, then incubated overnight in a 37°C humid chamber with CREST (Fisher; human CREST antiserum) and MLH1 (BC Pharmingen; mouse anti-human) diluted 1:500 and 1:75 respectively, or RAD51 (Santa Cruz; rabbit anti-human) diluted 1:75. SYCP3 (Novus; rabbit anti-human) at a 1:150 concentration was added and slides incubated for two hours, followed by 30 and 60 minute washes in 1x ADB. Secondary antibodies (Jackson ImmunoResearch) were added for overnight incubation; i.e., fluorescein anti-mouse (1:75) and AMCA anti-human (1:100) for MLH1 staining or fluorescein anti-rabbit (1:75) for RAD51 staining, followed by a 45 minute incubation with rhodamine anti-rabbit diluted 1:100, and two final washes in 1x PBS for 30 minutes and 60 minutes. Slides were fixed using Prolong Gold Antifade reagent (Invitrogen), sealed with rubber cement, and stored at 4°C until analysis.

Immunostaining for simultaneous visualization of SYCP1 and SYCP3 followed a slightly modified protocol. After blocking in 1x ADB for one hour, slides were incubated overnight in a 37°C humid chamber with CREST (Fisher; human CREST antiserum) and SYCP1 (Santa Cruz; goat anti-human) diluted 1:500 and 1:150 respectively. Slides were pre-washed in 1x ADB for 30 minutes and 60 minutes, followed by a two-hour incubation with SYCP3 (Novus; rabbit anti-human) at a 1:150 concentration. Slides were washed in 1x ADB for 30 minutes and 60 minutes, then incubated for two hours with secondary antibodies rhodamine anti-goat (1:150) and AMCA anti-human (1:100) (Jackson ImmunoResearch), followed by 30 and 60 minute washes in 1x PBS. Slides were then incubated with secondary antibody fluorescein anti-rabbit (Jackson ImmunoResearch) diluted 1:100 for 45 minutes, washed in 1x PBS for 45 minutes, and washed in 1x PBS overnight in the dark. Slides were fixed using Prolong Gold Antifade reagent (Invitrogen), sealed with rubber cement, and stored at 4°C until analysis. Slides were imaged on a Zeiss epifluorecence microscope with coordinates noted for each immunostained cell for subsequent fluorescence in-situ hybridization (FISH) analysis.

### Fluorescence in-situ hybridization (FISH)

Chromosome-specific FISH probes for chromosomes 1, 6, 9, 13, 14, 15, 16, 18, 21, and 22 were applied to slides previously immunostained for MLH1. Chromosome-specific probes consisted of Telomere 1q SpectrumGreen, Telomere 6q SpectrumOrange, Telomere 9p SpectrumGreen, Telomere 15q SpectrumOrange, WCP 16 SpectrumGreen, and CEP 18 alpha SpectrumGreen (Vysis), and probes detecting pericentromeric regions on chromosomes 13 and 21, or 14 and 22 (MP Biomedicals). Stained slides were dehydrated in a series of ethanol baths (75%, 90%, and 100%) before being denatured in 70% formamide/2x SSC for 5 minutes at 73°C and dehydrated again in the same ethanol series. The hybridization mix (30μl LSI buffer, 8μl H_2_O, and 1μl probe for chromosomes 1, 6, 9, 15, 16 or 18; 24μl buffer and 8μl probe for chromosomes 13 and 21 or 14 and 22) was denatured at 73°C for 5 minutes, and then kept at 37°C until ready. The hybridization mix was added to each slide before being incubated overnight at 37°C in a humid chamber. The slides were then washed in 0.4x SSC for 10 seconds, 2x SSC/0.1% NP-40 for 3 seconds, rinsed in distilled water, and dehydrated in an ethanol series (70%, 90%, and 100%). Slides were then air-dried, fixed with Prolong Gold Antifade reagent with DAPI (Invitrogen), and stored at 4°C until analysis.

Chromosome-specific DNA loop sizes were examined with whole-chromosome paint probes (AquariusCy3 chromosome 1, Aquarius FITC chromosome 16 and Aquarius Cy3 chromosome 21; Cytocell), using procedures described by Novak et al. [61]. Slides were washed in 2x SSC for 2 minutes and dehydrated in ethanol washes (70%, 85%, 100%) for 2 minutes each, and 20μL probe was placed on each slide. Coverslips were added, sealed with rubber cement, and placed on to a 75°C hotplate for 2 minutes to denature. They were incubated at 37°C overnight, washed in 0.4x SSC at 72°C for 2 minutes, drained and washed in 2x SSC/0.05% Tween20 at room temperature for 30 seconds, drained, Prolong Gold Antifade reagent with DAPI (Invitrogen) applied, and the slides stored at 4°C. Cells that had been previously identified for MLH1 or SYCP1 immunostaining were re-located and the FISH images captured.

### Cytological analysis

Pachytene stage cells were analyzed for the number and location of MLH1 foci. Foci were counted only if the MLH1 signals co-localized with SYCP3 signals, were punctate in appearance and were separated from adjacent MLH1 foci by at least one signal domain. All cells were scored by at least two observers.

For analysis of RAD51 foci, we analyzed leptotene or zygotene cells, scoring only those signals that were clearly located within the nuclear boundary (as indicated by the presence of DAPI staining). The number of foci per cell was calculated as the average of two scores from two independent observers.

### Analysis of SC length, DNA loop size, and CO spacing

MicroMeasure 3.3 [62] was used to measure synaptonemal complex length (taken as the length of the SYCP3 signal) in fully synapsed pachytene cells. We made genome-wide SC measurements and, in addition, analyzed ten representative chromosomes (1, 6, 9, 13, 14, 15, 16, 18, 21, and 22), identified by FISH paint probes. DNA loop size was assayed on chromosomes 1, 16, and 21 by taking the averages of measurements of the width of the FISH signal perpendicular to the SC at various points along the SC. For chromosomes 1 and 16, measurements were made at the centromere and at three equally spaced points on each chromosome arm; for chromosome 21, one measurement was made at the centromere and two equally spaced points on the long arm.

Crossover spacing was determined as the distance between adjacent MLH1 foci along the SC for the same ten representative chromosomes. Chromosomes with exactly four MLH1 foci were analyzed for chromosome 1 and chromosomes with exactly two MLH1 foci were measured for chromosomes 6, 9, 13, 14, 15, 16, 18, 21, and 22. Each inter-focal distance was then calculated as a percentage of the total SC length.

## Supporting Information

Figure S1 **Chromatin compaction in leptotene spermatocytes and oocytes.** Leptotene cells of both sexes were identified by the presence of multiple, short SYCP3 positive fragments (images not shown). Subsequently, slides were denatured and re-hybridized with a chromosome 16-specific FISH paint probe to visualize chromatin morphology of an individual chromosome. (A) Representative images of a leptotene spermatocyte and (B) a leptotene oocyte. In general, chromosome 16 FISH signals were longer and less widely dispersed in females.
(TIF)

Table S1 **Summary of patient information on 56 adult testicular biopsy samples.**
(DOCX)

Table S2 **Summary of MLH1 analyses of 56 adult testicular biopsy samples.**
(DOCX)

Table S3 **Summary of patient information on 63 fetal ovarian samples.**
(DOCX)

Table S4 **Summary of MLH1 analyses from 63 fetal ovarian samples.**
(DOCX)

Table S5 **Chromosome specific MLH1 exchange distribution.**
(DOCX)
