# Supplementary figures and images for: Cytological Studies of Human Meiosis: Sex-Specific Differences in Recombination Originate at, or Prior to, Establishment of Double-Strand Breaks
Source: PLoS One. 2013 Dec 20;8(12):e85075. doi: 10.1371/journal.pone.0085075 (PMC3869931; doi:10.1371/journal.pone.0085075)

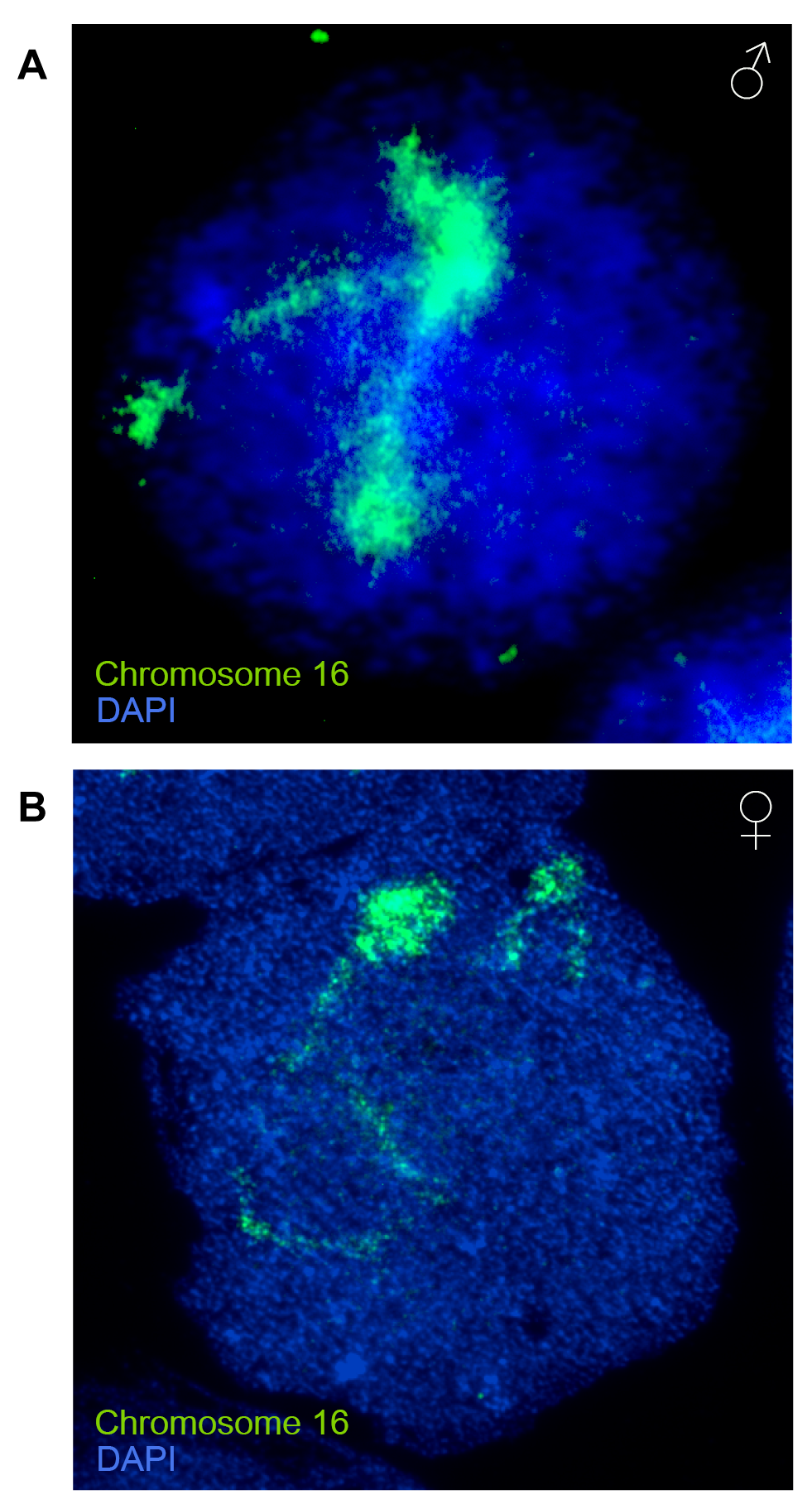

Supplement: Figure S1 — Chromatin compaction in leptotene spermatocytes and oocytes. Leptotene cells of both sexes were identified by the presence of multiple, short SYCP3 positive fragments (images not shown). Subsequently, slides were denatured and re-hybridized with a chromosome 16-specific FISH paint probe to visualize chromatin morphology of an individual chromosome. (A) Representative images of a leptotene spermatocyte and (B) a leptotene oocyte. In general, chromosome 16 FISH signals were longer and less widely dispersed in females. (TIF) [file pone.0085075.s001.tif]
