# Supplementary material for: Cytological Studies of Human Meiosis: Sex-Specific Differences in Recombination Originate at, or Prior to, Establishment of Double-Strand Breaks
Source: PLoS One. 2013 Dec 20;8(12):e85075. doi: 10.1371/journal.pone.0085075 (PMC3869931; doi:10.1371/journal.pone.0085075)
Supplement: Table S1 — Summary of patient information on 56 adult testicular biopsy samples. (DOCX) [file pone.0085075.s002.docx]

| **Table S1.**  Summary of patient information on 56 adult testicular biopsy samples. | | | | |
| --- | --- | --- | --- | --- |
|  |  |  |  |  |
|  | **ID** | **Age** | **Diagnosis** | **Chromosome**  **Constitution** |
| * | Sp319 | 31 | Oligospermia |  |
| * | Sp338 | 36 | Previous Vasectomy |  |
| * | Sp341 | 78 |  | 46,XY |
| * | Sp345 | 32 | Cystic Fibrosis | 46,XY |
| * | Sp350 | 31 | Varicocele |  |
| * | Sp357 | 33 | Severe oligospermia | 46,XY |
| * | Sp362 | 44 | Previous Vasectomy | 46,XY |
| * | Sp363 | 73 | Prostate cancer |  |
| * | Sp364 | 41 | Previous Vasectomy | 46,XY |
| * | Sp366 | 32 | Cystic Fibrosis | 46,XY |
| * | Sp367 | 45 | Previous Vasectomy |  |
| * | Sp368 | 30 | Cystic Fibrosis | 46,XY |
| * | Sp370 | 33 | Previous Vasectomy | 46,XY |
| * | Sp371 | 44 | Previous Vasectomy |  |
| * | Sp372 | 40 | Previous Vasectomy | 46,XY |
| * | Sp376 | 46 | Previous Vasectomy |  |
| * | Sp377 | 34 | Previous Vasectomy | 46,XY |
| * | Sp379 | 31 |  | 46,XY |
| * | Sp382 | 48 | Previous Vasectomy | 46,XY |
| * | Sp383 | 21 |  | 46,XY |
| * | Sp393 | 31 | Cystic Fibrosis | 46,XY |
|  | Sp401 | 43 | Previous Vasectomy | 46,XY |
| * | Sp402 | 41 | Previous Vasectomy | 46,XY |
| * | Sp403 | 31 | Previous Vasectomy | 46,XY |
|  | Sp404 | 34 | Cystic Fibrosis |  |
|  | Sp405 | 34 | Seminoma testicular mass | 46,XY |
| * | Sp407 | 26 | Cystic Fibrosis | 46,XY |
| * | Sp410 | 30 | Cystic Fibrosis | 46,XY |
|  | Sp413 | 60 | Previous Vasectomy | 46,XY |
|  | Sp414 | 44 | Previous Vasectomy | 46,XY |
|  | Sp1005 | 51 | Previous Vasectomy |  |
|  | Sp1006 | 39 |  |  |
|  | Sp2016 |  | Previous Vasectomy |  |
|  | OA-1 | 47 | Previous Vasectomy |  |
|  | OA-2 | 60 | Previous Vasectomy |  |
|  | OA-3 | 38 | Previous Vasectomy |  |
|  | OA-4 | 53 | Previous Vasectomy |  |
|  | OA-5 | 32 | Previous Vasectomy |  |
|  | OA-6 | 44 | Previous Vasectomy |  |
|  | OA-7 | 31 | Previous Vasectomy |  |
|  | OA-9 | 42 | Previous Vasectomy |  |
|  | OA-12 | 47 | Previous Vasectomy |  |
|  | OA-13 | 35 | Previous Vasectomy |  |
|  | OA-14 | 44 | Previous Vasectomy |  |
|  | OA-15 | 51 | Previous Vasectomy |  |
|  | OA-16 | 42 | Previous Vasectomy |  |
|  | OA-17 | 60 | Previous Vasectomy |  |
|  | OA-18 | 49 | Previous Vasectomy |  |
|  | OA-19 | 49 | Previous Vasectomy |  |
|  | OA-20 | 48 | Previous Vasectomy |  |
|  | OA-21 | 35 | Previous Vasectomy |  |
|  | OA-22 | 46 | Previous Vasectomy |  |
|  | OA-23 | 47 | Previous Vasectomy |  |
|  | OA-24 | 54 | Previous Vasectomy |  |
|  | OA-25 | 54 | Previous Vasectomy |  |
|  | OA-26 | 50 | Previous Vasectomy |  |

*previously reported in [23]
