# Supplementary material for: Cytological Studies of Human Meiosis: Sex-Specific Differences in Recombination Originate at, or Prior to, Establishment of Double-Strand Breaks
Source: PLoS One. 2013 Dec 20;8(12):e85075. doi: 10.1371/journal.pone.0085075 (PMC3869931; doi:10.1371/journal.pone.0085075)
Supplement: Table S2 — Summary of MLH1 analyses of 56 adult testicular biopsy samples. (DOCX) [file pone.0085075.s003.docx]

| **Table S2.** Summary of MLH1 analyses of 56 adult testicular biopsy samples. | | | | |
| --- | --- | --- | --- | --- |
|  |  |  |  |  |
|  | ID | Number of Cells | Mean MLH1 ± S.E. | Range |
| * | Sp319 | 65 | 49.4 ± 0.6 | 39-57 |
| * | Sp338 | 60 | 51.9 ± 0.5 | 41-60 |
| * | Sp341 | 39 | 49.5 ± 0.7 | 42-58 |
| * | Sp345 | 38 | 48.5 ± 0.6 | 43-57 |
| * | Sp350 | 20 | 50.2 ± 0.7 | 45-57 |
| * | Sp357 | 79 | 51.3 ± 0.6 | 38-64 |
| * | Sp362 | 74 | 46.8 ± 0.5 | 36-59 |
| * | Sp363 | 68 | 52.8 ± 0.6 | 42-66 |
| * | Sp364 | 84 | 46.2 ± 0.4 | 39-53 |
| * | Sp366 | 132 | 49.7 ± 0.3 | 42-59 |
| * | Sp367 | 208 | 50.4 ± 0.3 | 36-64 |
| * | Sp368 | 112 | 48.8 ± 0.5 | 39-63 |
| * | Sp370 | 238 | 46.6 ± 0.3 | 34-57 |
| * | Sp371 | 33 | 47.6 ± 0.9 | 37-57 |
| * | Sp372 | 169 | 49.4 ± 0.3 | 38-61 |
| * | Sp376 | 62 | 50.3 ± 0.5 | 40-60 |
| * | Sp377 | 62 | 49.2 ± 0.7 | 38-60 |
| * | Sp379 | 70 | 47.4 ± 0.5 | 35-58 |
| * | Sp382 | 108 | 48.1 ± 0.4 | 37-60 |
| * | Sp383 | 49 | 48.7 ± 0.6 | 39-59 |
| * | Sp393 | 95 | 54.1 ± 0.4 | 45-62 |
|  | Sp401 | 85 | 48.7 ± 0.5 | 37-58 |
| * | Sp402 | 75 | 50.0 ± 0.4 | 40-60 |
| * | Sp403 | 150 | 50.0 ± 0.4 | 30-61 |
|  | Sp404 | 145 | 45.1 ± 0.3 | 35-56 |
|  | Sp405 | 64 | 46.1 ± 0.4 | 39-58 |
| * | Sp407 | 89 | 54.3 ± 0.5 | 35-66 |
| * | Sp410 | 29 | 52.7 ± 0.9 | 46-62 |
|  | Sp413 | 157 | 45.7 ± 0.3 | 35-57 |
|  | Sp414 | 73 | 51.5 ± 0.5 | 44-61 |
|  | Sp1005 | 120 | 53.7 ± 0.4 | 42-65 |
|  | Sp1006 | 97 | 48.1 ± 0.4 | 38-56 |
|  | Sp2016 | 105 | 47.8 ± 0.4 | 39-57 |
|  | OA-1 | 83 | 50.5 ± 0.4 | 44-61 |
|  | OA-2 | 9 | 46.0 ± 0.9 | 43-51 |
|  | OA-3 | 20 | 48.1 ± 0.9 | 43-56 |
|  | OA-4 | 42 | 46.6 ± 0.5 | 41-54 |
|  | OA-5 | 177 | 52.7 ± 0.3 | 37-64 |
|  | OA-6 | 44 | 43.3 ± 0.4 | 39-48 |
|  | OA-7 | 95 | 48.0 ± 0.3 | 40-57 |
|  | OA-9 | 80 | 50.6 ± 0.5 | 41-61 |
|  | OA-12 | 138 | 44.6 ± 0.3 | 38-53 |
|  | OA-13 | 49 | 47.8 ± 0.5 | 42-59 |
|  | OA-14 | 46 | 50.2 ± 0.5 | 42-58 |
|  | OA-15 | 161 | 47.3 ± 0.3 | 39-56 |
|  | OA-16 | 95 | 47.9 ± 0.4 | 39-56 |
|  | OA-17 | 149 | 49.5 ± 0.3 | 41-62 |
|  | OA-18 | 173 | 52.2 ± 0.4 | 40-65 |
|  | OA-19 | 45 | 46.7 ± 0.5 | 41-53 |
|  | OA-20 | 42 | 49.5 ± 0.7 | 39-60 |
|  | OA-21 | 40 | 48.9 ± 0.6 | 43-60 |
|  | OA-22 | 23 | 52.9 ± 0.7 | 49-59 |
|  | OA-23 | 18 | 49.1 ± 0.8 | 43-56 |
|  | OA-24 | 37 | 47.7 ± 0.7 | 40-57 |
|  | OA-25 | 9 | 48.4 ± 1.1 | 44-54 |
|  | OA-26 | 31 | 47.6 ± 0.7 | 39-54 |
|  | **Total** | **4660** | **49.1 ± 0.1** | **30-66** |

*previously reported in [23]
