# Supplementary material for: Cytological Studies of Human Meiosis: Sex-Specific Differences in Recombination Originate at, or Prior to, Establishment of Double-Strand Breaks
Source: PLoS One. 2013 Dec 20;8(12):e85075. doi: 10.1371/journal.pone.0085075 (PMC3869931; doi:10.1371/journal.pone.0085075)
Supplement: Table S3 — Summary of patient information on 63 fetal ovarian samples. (DOCX) [file pone.0085075.s004.docx]

| **Table S3.**  Summary of patient information on 63 fetal ovarian samples. | | | | | |
| --- | --- | --- | --- | --- | --- |
|  |  |  |  |  |  |
|  | **ID** | **Maternal Age** | **Gestational Age (weeks)** | **Diagnosis** | **Chromosome Constitution** |
|  | EC0006 |  | 21 0/7 |  | 46, XX |
| * | EC0010 |  | 18 2/7 |  | 46, XX |
| * | EC0018 |  | 22 0/7 |  | 46, XX |
| * | EC0041 |  | 16 0/7 | Anencephaly |  |
| * | EC0053 |  | 16 0/7 |  |  |
| * | EC0069 |  | 19 6/7 | Multiple congenital anomalies | 46, XX |
| * | EC0076 |  | 19 1/7 | Neurotube defect |  |
| * | EC0091 |  | 21 2/7 |  |  |
| * | EC0096 |  | 20 3/7 | Renal agensis, anhydramnios | 46, XX |
| * | EC0098 |  | 17 4/7 | Anencephaly |  |
| * | EC0099 |  | 19 0/7 |  |  |
| * | EC0101 |  | 20 0/7 | Maternal vaginal lymphoma |  |
| * | EC0141 |  | 15 0/7 |  |  |
| * | EC0143 |  | 14 0/7 |  |  |
| * | EC0147 |  | 18 0/7 | Placenta accreta |  |
|  | EC0174 |  | 19 2/7 |  |  |
| * | SF0001 | 21 | 22 1/7 | Elective termination |  |
| * | SF0002 | 21 | 22 1/7 | Elective termination |  |
| * | SF0004 | 27 | 21 2/7 | Elective termination |  |
| * | SF0008 | 25 | 21 0/7 | Elective termination |  |
| * | SF0009 | 31 | 23 5/7 | Elective termination |  |
| * | SF0010 | 30 | 20 1/7 | Elective termination |  |
| * | SF0011 | 18 | 25 1/7 | Elective termination |  |
| * | SF0012 | 29 | 24 3/7 | Elective termination |  |
| * | SF0013 | 21 | 16 3/7 | Elective termination |  |
| * | SF0018 | 32 | 25 1/7 | Elective termination |  |
| * | SF0020 | 31 | 21 3/7 | Elective termination |  |
| * | SF0023 | 18 | 23 2/7 | Elective termination |  |
| * | SF0024 | 37 | 19 0/7 | Elective termination |  |
| * | SF0025 | 21 | 19 6/7 | Elective termination |  |
| * | SF0029 | 16 | 24 2/7 | Elective termination |  |
| * | SF0032 | 24 | 22 2/7 | Elective termination |  |
| * | SF0035 | 19 | 20 3/7 | Elective termination |  |
|  | SF0039 |  | 21 3/7 | Elective termination |  |
|  | SF0040 |  | 22 4/7 | Elective termination |  |
|  | SF0042 |  | 20 4/7 | Elective termination |  |
|  | SF0046 |  | 18 5/7 | Elective termination |  |
|  | SF0047 |  | 22 3/7 | Elective termination |  |
|  | SF0048 |  | 20 0/7 | Elective termination |  |
|  | SF0049 |  | 20 0/7 | Elective termination |  |
|  | SF0053 |  | 20 0/7 | Elective termination |  |
|  | SF0054 |  | 22 0/7 | Elective termination |  |
|  | SF0055 |  | 22 6/7 | Elective termination |  |
|  | SF0056 |  | 24 6/7 | Elective termination |  |
|  | SF0057 |  | 16 6/7 | Elective termination |  |
|  | SF0059 |  | 20 4/7 | Elective termination |  |
|  | SF0060 |  | 21 4/7 | Elective termination |  |
|  | SF0063 |  | 21 0/7 | Elective termination |  |
|  | SF0065 |  | 20 2/7 | Elective termination |  |
|  | SF0067 |  | 23 5/7 | Elective termination |  |
|  | SF0069 |  | 17 3/7 | Elective termination |  |
|  | SF0070 |  | 18 5/7 | Elective termination |  |
|  | SF0072 |  | 17 3/7 | Elective termination |  |
|  | SF0073 |  | 16 4/7 | Elective termination |  |
|  | SF0075 |  | 21 6/7 | Elective termination |  |
|  | SF0076 |  | 18 6/7 | Elective termination |  |
|  | SF0077 |  | 20 0/7 | Elective termination |  |
|  | SF0078 |  | 22 1/7 | Elective termination |  |
|  | SF0082 |  | 21 1/7 | Elective termination |  |
|  | SF0083 |  | 22 2/7 | Elective termination |  |
|  | SF0084 |  | 20 0/7 | Elective termination |  |
|  | SF0085 |  | 19 6/7 | Elective termination |  |
|  | SF0086 |  | 19 2/7 | Elective termination |  |

*previously reported in [21]
