# Supplementary material for: Cytological Studies of Human Meiosis: Sex-Specific Differences in Recombination Originate at, or Prior to, Establishment of Double-Strand Breaks
Source: PLoS One. 2013 Dec 20;8(12):e85075. doi: 10.1371/journal.pone.0085075 (PMC3869931; doi:10.1371/journal.pone.0085075)
Supplement: Table S4 — Summary of MLH1 analyses from 63 fetal ovarian samples. (DOCX) [file pone.0085075.s005.docx]

| **Table S4.** Summary of MLH1 analyses from 63 fetal ovarian samples | | | | |
| --- | --- | --- | --- | --- |
|  |  |  |  |  |
|  | **ID** | **Number of Cells** | **Mean MLH1 ± S.D.** | **Range** |
|  | EC0006 | 5 | 74.6 ± 5.2 | 56-86 |
| * | EC0010 | 25 | 59.3 ± 2.0 | 43-81 |
| * | EC0018 | 7 | 80.7 ± 6.8 | 53-96 |
| * | EC0041 | 71 | 65.1 ± 1.4 | 40-89 |
| * | EC0053 | 14 | 69.8 ± 3.3 | 46-89 |
| * | EC0069 | 40 | 68.2 ± 1.4 | 44-83 |
| * | EC0076 | 36 | 71.7 ± 1.8 | 54-95 |
| * | EC0091 | 39 | 68.6 ± 1.5 | 49-92 |
| * | EC0096 | 60 | 79.6 ± 1.4 | 59-102 |
| * | EC0098 | 30 | 82.1 ± 1.9 | 57-107 |
| * | EC0099 | 37 | 87.1 ± 2.6 | 50-115 |
| * | EC0101 | 53 | 88.3 ± 1.4 | 66-109 |
| * | EC0141 | 13 | 76.3 ± 3.9 | 53-95 |
| * | EC0143 | 11 | 87.2 ± 3.3 | 64-104 |
| * | EC0147 | 40 | 67.8 ± 1.7 | 48-88 |
|  | EC0174 | 34 | 69.9 ± 2.4 | 46-98 |
| * | SF0001 | 59 | 59.2 ± 1.8 | 27-90 |
| * | SF0002 | 34 | 59.7 ± 1.8 | 37-83 |
| * | SF0004 | 17 | 59.1 ± 3.4 | 40-96 |
| * | SF0008 | 70 | 66.6 ± 1.4 | 42-99 |
| * | SF0009 | 13 | 52.6 ± 2.9 | 40-77 |
| * | SF0010 | 54 | 65.5 ± 1.7 | 45-100 |
| * | SF0011 | 10 | 75.0 ± 2.8 | 55-89 |
| * | SF0012 | 10 | 64.8 ± 2.5 | 52-76 |
| * | SF0013 | 40 | 66.4 ± 2.2 | 43-97 |
| * | SF0018 | 12 | 72.3 ± 4.2 | 56-107 |
| * | SF0020 | 11 | 60.2 ± 3.3 | 48-83 |
| * | SF0023 | 20 | 64.3 ± 2.8 | 45-86 |
| * | SF0024 | 39 | 61.8 ± 1.7 | 45-90 |
| * | SF0025 | 46 | 59.9 ± 1.4 | 43-83 |
| * | SF0029 | 18 | 69.6 ± 2.6 | 55-92 |
| * | SF0032 | 50 | 73.3 ± 1.6 | 51-103 |
| * | SF0035 | 56 | 69.9 ± 1.4 | 50-100 |
|  | SF0039 | 11 | 63.1 ± 2.5 | 56-82 |
|  | SF0040 | 14 | 63.3 ± 3.1 | 42-80 |
|  | SF0042 | 18 | 73.7 ± 2.9 | 45-103 |
|  | SF0046 | 65 | 60.9 ± 1.3 | 41-88 |
|  | SF0047 | 26 | 74.4 ± 2.2 | 60-98 |
|  | SF0048 | 21 | 57.9 ± 2.2 | 41-80 |
|  | SF0049 | 37 | 66.8 ± 1.8 | 46-87 |
|  | SF0053 | 57 | 71.5 ± 1.2 | 53-92 |
|  | SF0054 | 18 | 62.8 ± 3.1 | 42-95 |
|  | SF0055 | 17 | 66.0 ± 2.5 | 46-82 |
|  | SF0056 | 36 | 77.5 ± 2.1 | 54-105 |
|  | SF0057 | 17 | 65.4 ± 3.5 | 48-99 |
|  | SF0059 | 28 | 66.7 ± 2.1 | 43-92 |
|  | SF0060 | 11 | 72.3 ± 2.2 | 61-85 |
|  | SF0063 | 22 | 72.3 ± 2.5 | 46-95 |
|  | SF0065 | 10 | 65.9 ± 4.1 | 47-87 |
|  | SF0067 | 13 | 66.5 ± 3.3 | 51-88 |
|  | SF0069 | 22 | 64.2 ± 3.5 | 37-98 |
|  | SF0070 | 31 | 65.3 ± 1.8 | 44-85 |
|  | SF0072 | 11 | 65.9 ± 3.5 | 44-83 |
|  | SF0073 | 27 | 56.8 ± 1.7 | 36-74 |
|  | SF0075 | 6 | 71.9 ± 7.2 | 54-94 |
|  | SF0076 | 49 | 74.2 ± 1.6 | 54-107 |
|  | SF0077 | 5 | 69.7 ± 5.1 | 53-80 |
|  | SF0078 | 104 | 72.7 ± 1.3 | 48-119 |
|  | SF0082 | 65 | 73.4 ± 1.2 | 53-101 |
|  | SF0083 | 2 | 75.0 ± 15.0 | 60-90 |
|  | SF0084 | 62 | 69.9 ± 1.3 | 48-95 |
|  | SF0085 | 52 | 71.3 ± 1.4 | 51-94 |
|  | SF0086 | 107 | 70.5 ± 0.8 | 47-93 |
|  | **Total** | **2038** | **69.3 ± 0.29** | **27-119** |

*previously reported in [21]
