# Supplementary material for: Cytological Studies of Human Meiosis: Sex-Specific Differences in Recombination Originate at, or Prior to, Establishment of Double-Strand Breaks
Source: PLoS One. 2013 Dec 20;8(12):e85075. doi: 10.1371/journal.pone.0085075 (PMC3869931; doi:10.1371/journal.pone.0085075)
Supplement: Table S5 — Chromosome specific MLH1 exchange distribution. (DOCX) [file pone.0085075.s006.docx]

| **Table S5. Chromosome specific MLH1 exchange distribution** | | | | | | | | | | | | | | | | | | | | |  |
| --- | --- | --- | --- | --- | --- | --- | --- | --- | --- | --- | --- | --- | --- | --- | --- | --- | --- | --- | --- | --- | --- |
|  |  |  |  |  |  |  |  |  |  |  |  |  |  |  |  |  |  |  |  |  |  |
|  | | **Chromosome** | | | | | | | | | | | | | | | | | | | |
|  | | **1** | | **6** | | **9** | | **13** | | **14** | | **15** | | **16** | | **18** | | **21** | | **22** | |
|  | | M | F | M | F | M | F | M | F | M | F | M | F | M | F | M | F | M | F | M | F |
| E0 | |  |  |  |  |  |  |  | 1 |  | 1 |  |  |  | 1 |  | 3 | 4 | 14 | 4 | 11 |
| E1 | |  |  | 3 |  | 1 | 3 | 18 | 14 | 25 | 15 | 12 | 10 | 18 | 6 | 41 | 21 | 401 | 187 | 324 | 115 |
| E2 | | 12 | 1 | 78 | 6 | 29 | 10 | 117 | 68 | 107 | 26 | 110 | 16 | 214 | 51 | 143 | 73 | 16 | 74 | 100 | 81 |
| E3 | | 55 | 4 | 86 | 5 | 26 | 14 | 4 | 64 | 10 | 26 | 8 | 15 | 15 | 55 | 3 | 48 |  | 4 |  | 10 |
| E4 | | 95 | 15 | 7 | 12 | 2 | 4 |  | 21 |  | 2 |  | 3 |  | 5 |  | 12 |  |  |  |  |
| E5+ | | 23 | 63 |  | 7 |  | 3 |  | 3 |  |  |  | 1 |  | 2 |  | 1 |  |  |  |  |
| N | | 185 | 83 | 174 | 30 | 58 | 34 | 139 | 171 | 142 | 70 | 130 | 45 | 247 | 120 | 187 | 158 | 421 | 279 | 428 | 217 |
| Mean± S.E. | | 3.7±0.06 | 5.9±0.18 | 2.6±0.05 | 3.7±0.19 | 2.5±0.08 | 2.8±0.05 | 1.9±0.03 | 2.6±0.07 | 1.9±0.04 | 2.2±0.10 | 2.0±0.03 | 2.3±0.15 | 2.0±0.02 | 2.5±0.07 | 1.8±0.03 | 2.3±0.07 | 1.0±0.01 | 1.2±0.03 | 1.2±0.02 | 1.4±0.04 |
| M:F ratio | | 0.63 | | 0.70 | | 0.81 | | 0.73 | | 0.86 | | 0.87 | | 0.80 | | 0.78 | | 0.83 | | 0.86 | |
| Χ^2^ | | <0.0001 | | <0.0001 | | 0.02 | | <0.0001 | | 0.005 | | <0.0001 | | <0.0001 | | <0.0001 | | <0.0001 | | <0.0001 | |
